# ﻿Morphology and phylogeny of two new species within Cordycipitaceae (Hypocreales) from China

**DOI:** 10.3897/mycokeys.115.140683

**Published:** 2025-03-17

**Authors:** Yingling Lu, Songyu Li, Zuoheng Liu, Jing Zhao, Zhiyong Yu, Zongli Liang, Hailong He, Jianhong Li, Yun Huang, Xinming Li, Hong Yu

**Affiliations:** 1 Yunnan Herbal Laboratory, College of Ecology and Environmental Sciences, Yunnan University, Kunming, Yunnan 650504, China; 2 The International Joint Research Center for Sustainable Utilization of Cordyceps Bioresources in China and Southeast Asia, Yunnan University, Kunming 650091, China; 3 Yunnan Jinping Fenshuiling National Nature Reserve, Honghe, Yunnan 661500, China

**Keywords:** *Leptobacillium*, morphology, new taxa, phylogenetic analysis, *Simplicillium*, taxonomy

## Abstract

*Simplicillium* and *Leptobacillium*, sister genera in the family Cordycipitaceae, exhibit a broad range of hosts or substrates. The identification of two novel species, from *Simplicillium* and *Leptobacillium*, was achieved by analysing morphological characteristics and phylogenetic data obtained from six molecular markers (ITS, nrSSU, nrLSU, *tef-1α*, *rpb1* and *rpb2*). The two recently documented species are *S.puwenense* and *L.longiphialidum*. Morphologically, *S.puwenense* possessed slender solitary rod-shaped or columnar phialides with elliptical oval or cylindrical conidia forming small spherical heads at the apex of phialides. On the other hand, *L.longiphialidum* had solitary columnar phialides with elliptic or subspherical apical conidia while other conidia were narrow columnar or fusiform in shape. Phylogenetic analysis revealed that *S.puwenense* formed an independent branch as a sister species to *S.formicae*, whereas *L.longiphialidum* clustered with *L.marksiae* exhibiting stable topological structure. The Bayesian inference posterior probability and the maximum likelihood bootstrap-ratio provided robust statistical evidence, indicating the presence of two novel species within the genera of *Simplicillium* and *Leptobacillium*. The present study contributes to the discovery of species diversity in *Simplicillium* and *Leptobacillium*, while also providing a taxonomic foundation for their rational development and sustainable utilisation.

## ﻿Introduction

As was well known, many species in the family Cordycipitaceae Kreisel ex G.H. Sung, Hywel-Jones & Spatafora were entomogenous (Mongkolsamrit et al. 2018; Wang et al. 2020). Amongst them, the genera *Simplicillium* W. Gams & Zare and *Leptobacillium* Zare & W. Gams were sister genera (Zare and Gams 2001, 2016).

In 2001, Zare and Gams established the genus *Simplicillium*, which included *S.lanosoniveum* (J.F.H. Beyma) Zare & W. Gams (type species), *S.lamellicola* (F.E.V. Sm.) Zare & W. Gams, *S.obclavatum* (W. Gams) Zare & W. Gams and *S.wallacei* H.C. Evans. The key distinguishing characteristic of the *Simplicillium* genus was the solitary presence of phialides, with conidia typically adhering to the apex of phialides in chains that resemble spherical, sticky or tile-like structures, ultimately forming octahedral crystals (Zare and Gams 2001). The solitary phialides enabled the distinction between the genus *Simplicillium* and its closely-related genus *Lecanicillium* W. Gams & Zare (Chen et al. 2021). Species belonging to the *Simplicillium* genus exhibited ecological diversity, including their presence in various environments, such as soil, endophytic fungi of plants, rocks and decaying wood (Liu and Cai 2012; Nonaka et al. 2013; Zhang et al. 2017; Crous et al. 2018, 2021). *S.chinense* F. Liu & L. Cai was the first *Simplicillium* species discovered in China (Liu and Cai 2012).

In 2016, the genus *Leptobacillium* was established by Zare and Gams during the revision of the former Verticillium Nees section Albo-erecta. The name of the genus referred to its characteristic narrow microconidia, with the model species being *L.leptobactrum* (W. Gams) Zare & W. Gams (Zare and Gams 2016). The genus *Leptobacillium* comprised species that exhibited two distinct types of conidia. Individual cells aggregated to form chains, with nearly spherical or elliptical conidia located at the apex of long chains, while narrow cylindrical (rod-shaped) to fusiform conidia were found elsewhere within the chain (Zare and Gams 2016; Leplat et al. 2022). Zare and Gams (2016) initially described *L.leptobactrum*, a species consisting of two varieties, namely L.leptobactrumvar.leptobactrum (W. Gams) Zare & W. Gams and L.leptobactrumvar.calidius Zare & W. Gams, which were distinguished by their optimal growth temperatures. The species of *Leptobacillium* exhibited a wide range of host and substrate diversity, having been isolated from various sources including Lepidoptera insects, fungi, plants, fresh water, murals and rocks (Liu and Cai 2012; Zare and Gams 2016; Gomes et al. 2018; Crous et al. 2018; Sun et al. 2019; Okane et al. 2020). The nematophagous properties of *Leptobacillium* species have been extensively studied (Regaieg et al. 2011; Leplat et al. 2022).

Phylogenetic studies of species in the genera *Simplicillium* and *Leptobacillium* have focused on the nuclear ribosomal internal transcribed spacer region (ITS) and the nuclear ribosomal large subunit (nrLSU). Currently, several other DNA loci are frequently used to study species in the Cordycipitaceae family (Kepler et al. 2017; Wang et al. 2020; Leplat et al. 2022). Based on a phylogenetic analysis, *S.wallacei* was transplanted into the genus *Lecanicillium* and later Zhang et al. placed *L.wallacei* in the genus *Gamszarea* Z.F. Zhang & L. Cai (Zare and Gams 2001, 2008; Zhang et al. 2021). Phylogenetic analysis, based on five locus data, showed that *S.coffeanum* A.A.M. Gomes & O.L. Pereira, *S.chinensis* F. Liu & L. Cai and *S.filiforme* R.M.F. Silva, R.J.V. Oliveira, Souza-Motta, J.L. Bezerra & G.A. Silva were transferred to the genus *Leptobacillium* (Zare and Gams 2008; Okane et al. 2020; Chen et al. 2021).

Based on a comparative analysis of morphological characteristics and a multi-gene molecular phylogeny, we characterised in this study two newly-identified species from China, namely *S.puwenense* Hong Yu bis, Y.L. Lu & J. Zhao, sp. nov., from the genus of *Simplicillium* and *L.longiphialidum* Hong Yu bis, Y.L. Lu & J. Zhao, sp. nov., from the genus of *Leptobacillium*, respectively. This investigation has contributed to the expansion of the species diversity within the genera of *Simplicillium* and *Leptobacillium*, providing a solid taxonomic foundation to facilitate the rational development and sustainable use of these valuable resources.

## ﻿Material and method

### ﻿Material collection and isolation

The specimens of a dead spider infected with fungi were collected in China. One specimen was collected from Puwen Town, Jinghong City, Xishuangbanna Dai Autonomous Prefecture, Yunnan Province, China and the Xilong Mountains in Jinping County, Honghe Hani and Yi Autonomous Prefecture, Yunnan Province, China. Another was collected from Limushan National Forest Park, Limushan Town, Qiongzhong City, Hainan Province, China and 511 Township Road, Boluo County, Huizhou City, Guangdong Province, China. The specimens were photographed, assigned numbers and their collection details including habitat, elevation, latitude and longitude were documented. Subsequently, they were placed in freezing tubes within a vehicle-mounted refrigerator set at 4 °C for transportation back to the laboratory. Upon arrival at the laboratory, the specimens underwent initial observation and measurement using an Optec SZ660 stereo dissecting microscope. A select number of fungal conidia were then carefully picked with an inoculation needle and inoculated into PDA solid medium containing 0.05 g tetracycline and 0.1 g streptomycin using the plate streak method (Wang et al. 2020). The pure culture was incubated at a temperature of 25 °C, while the purified strain was transferred to a bevelled test tube containing PDA medium and stored at 4 °C (Wang et al. 2020). The specimens were deposited in the Yunnan Herbal Herbarium (YHH), while the strains were conserved in the Yunnan Fungal Culture Collection (YFCC).

### ﻿Morphological observations

The pure cultures were transferred to PDA solid medium and incubated at 25 °C for 14 days. Colony diameters were measured, colony characteristics were recorded and photographs of the front and back of the colonies were captured using a Canon camera (Tokyo, Japan). To observe the microscopic morphology of the colonies, filter paper was cut to fit a petri dish and placed inside. A U-shaped glass shelf, a slide and two coverlids that had been sterilised at 121 °C for 30 minutes and then dried were prepared. A layer of PDA medium with a thickness of 1 mm and size of approximately 5 mm was applied onto the slide. A small amount of mycelia was selected from each culture and transferred to the centre of the medium. It was covered with a coverslip, sterile water was added to moisten the medium and sealed in an incubator at 25 °C for cultivation. The microstructure was observed, measured and photographed using fluorescence microscopes CX40 (Tokyo, Japan) and BX53 (Tokyo, Japan).

### ﻿DNA extraction, polymerase chain reaction (PCR) and sequencing

The total genomic DNA of fungi was extracted using the CTAB method described by Liu et al. (2001). The ITS region was amplified using primer pairs ITS4 and ITS5 (White et al. 1990). The nuclear ribosomal small subunit (nrSSU) and nrLSU were amplified using primer pairs nrSSU-CoF with nrSSU-CoR and LR5 with LR0R, respectively (Vilgalys and Hester 1990; Rehner and Samuels 1994; Wang et al. 2015b). The translation elongation factor 1α (*tef-1α*) was amplified using primer pairs EF1α-EF and EF1α-ER (Bischoff et al. 2006; Sung et al. 2007). Finally, the largest subunit of RNA polymerase II (*rpb1*) and the second largest subunit of RNA polymerase II (*rpb2*) were amplified using primer pairs RPB1-5’F with RPB1-5’R and RPB2-5’F with RPB2-5’R, respectively, as described by Bischoff et al. (2006) and Sung et al. (2007).

The final volume of all PCR reactions was 25 µl, consisting of 17.25 µl of sterile deionised water, 2.5 µl of PCR10 Buffer (2 mmol/l Mg^2+^) from Transgen Biotech in Beijing, China, 2 µl of dNTP (2.5 mmol/l), 1 µl each of forward and reverse primers, 0.25 µl of Taq DNA polymerase from Transgen Biotech in Beijing, China and 1 µl of DNA template. The polymerase chain reaction (PCR) for the five genes and ITS was conducted using a BIO-RAD T100TM thermal cycler manufactured by BIO-RAD Laboratories in Hercules, CA, United States (Bischoff et al. 2006; Wang et al. 2015a). The PCR products were analysed through electrophoresis on a 1.0% agarose gel and subsequently stored at -20 °C until they were dispatched in dry ice to BGI Co., Ltd, Shenzhen, China for sequencing.

### ﻿Phylogenetic analyses

After aligning the six-gene sequences of related species obtained from GenBank with those of the present study using the Clustal W programme in MEGA v.5.0 software, we concatenated the six-gene datasets (ITS, nrSSU, nrLSU, *tef-1α*, *rpb1* and *rpb2*) into a combined matrix comprising all six genes. To both single gene and six-gene datasets, we respectively employed the ModelFinder programme in PhyloSuite v.1.2.2 software to determine the optimal model for the maximum likelihood analysis, based on Corrected AIC (AICc) and IQ-TREE model selection methods. The remaining parameters were set to their default values. Subsequently, we utilised the IQ-TREE programme with 5,000 bootstrap replicates to construct a maximum likelihood tree while selecting appropriate optimal model parameters.

The ModelFinder programme in PhyloSuite v.1.2.2 software was utilised to determine the optimal model for the Bayesian inference using Corrected AIC (AICc) and the MrBayes model, while keeping default settings for other parameters. Subsequently, the MrBayes programme was employed to select appropriate optimal model parameters and run for 2,000,000 generations to construct the BI tree. The constructed phylogenetic trees were visualised in FigTree v.1.4.2 to figure the maximum likelihood method of bootstrap proportion (BP) and the Bayesian inference posterior probability (BPP) and then formatted for editing with Adobe Illustrator CS6.

## ﻿Results

### ﻿Phylogenetic analyses

Phylogenetic analysis of single gene molecular fragments

**Figure 1.**
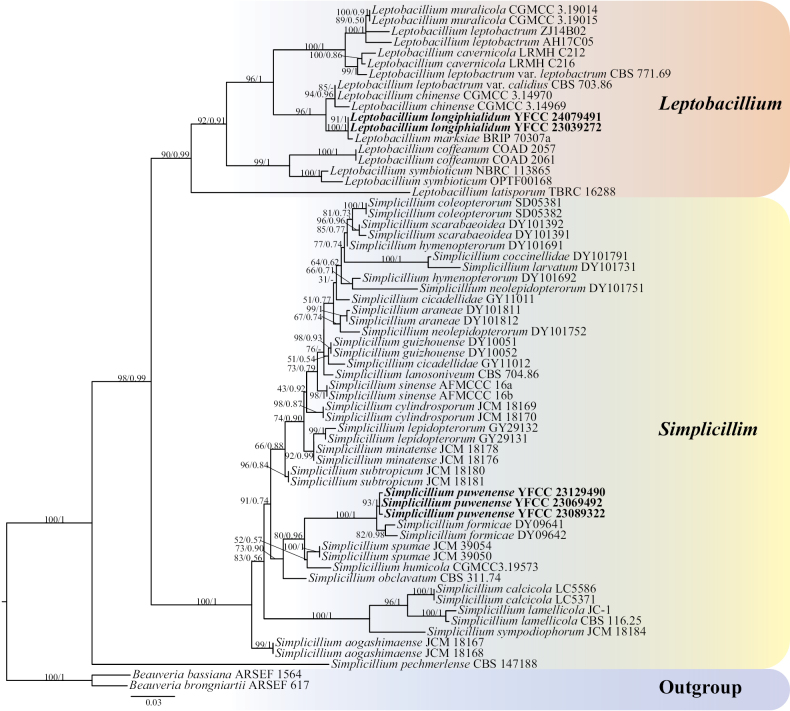
The phylogenetic tree of *Simplicillium* and *Leptobacillium* was inferred from ITS sequence, based on the Bayesian inference and the maximum likelihood analyses. Each value at a node indicates a bootstrap proportion (the left) and Bayesian posterior probability (the right). The scale bar 0.03 indicates the number of expected mutations per site. The species in bold black font of the *Simplicillium* and *Leptobacillium* were from this study. *B.bassiana* ARSEF 1564 and *B.brongniartii* ARSEF 617 were designated as outgroups.

**Figure 2.**
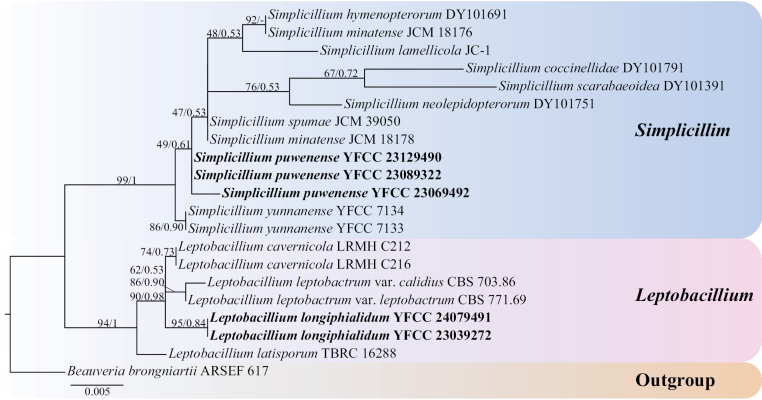
The phylogenetic tree of *Simplicillium* and *Leptobacillium* was inferred from nrSSU sequence, based on the Bayesian inference and the maximum likelihood analyses. Each value at a node indicates a bootstrap proportion (the left) and Bayesian posterior probability (the right). The scale bar 0.005 indicates the number of expected mutations per site. The species in bold black font of the *Simplicillium* and *Leptobacillium* were from this study. *B.brongniartii* ARSEF 617 was designated as outgroup.

**Figure 3.**
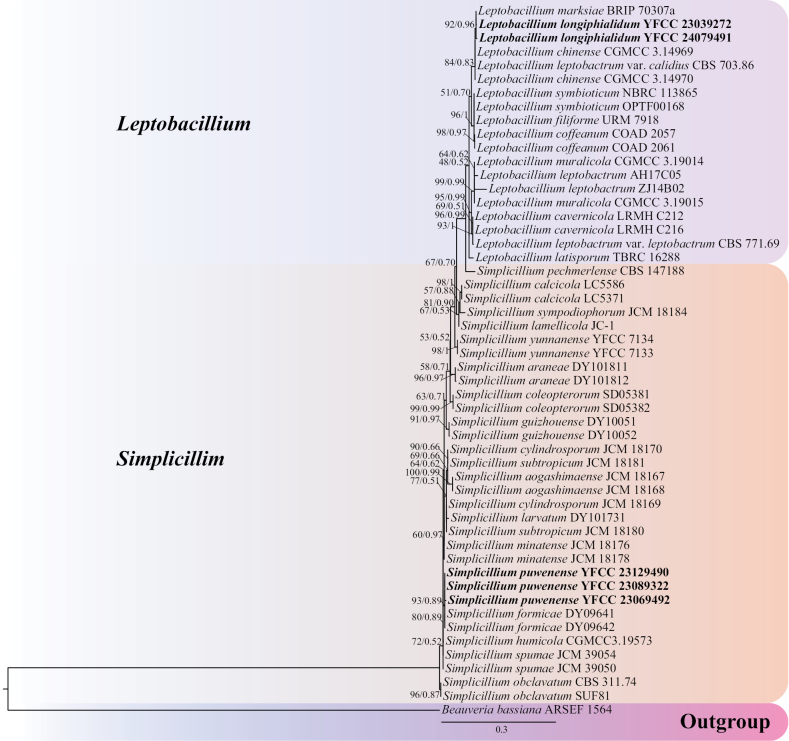
The phylogenetic tree of *Simplicillium* and *Leptobacillium* was inferred from nrLSU sequence, based on the Bayesian inference and the maximum likelihood analyses. Each value at a node indicates a bootstrap proportion (the left) and Bayesian posterior probability (the right). The scale bar 0.3 indicates the number of expected mutations per site. The species in bold black font of the *Simplicillium* and *Leptobacillium* were from this study. *B.bassiana* ARSEF 1564 was designated as outgroup.

**Figure 4.**
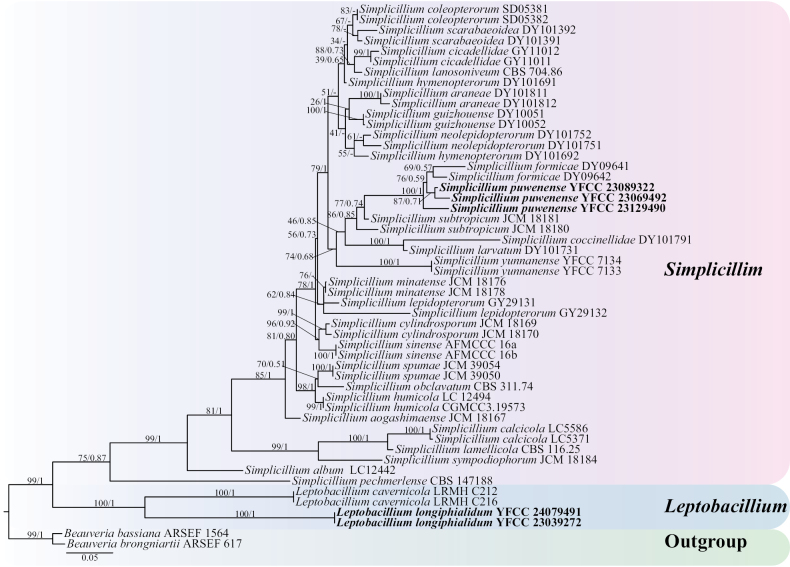
The phylogenetic tree of *Simplicillium* and *Leptobacillium* was inferred from *tef-1α* sequence, based on the Bayesian inference and the maximum likelihood analyses. Each value at a node indicates a bootstrap proportion (the left) and Bayesian posterior probability (the right). The scale bar 0.05 indicates the number of expected mutations per site. The species in bold black font of the *Simplicillium* and *Leptobacillium* were from this study. *B.bassiana* ARSEF 1564 and *B.brongniartii* ARSEF 617 were designated as outgroups.

**Figure 5.**
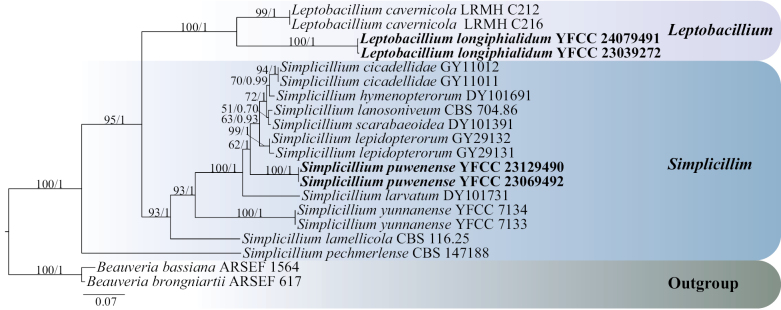
The phylogenetic tree of *Simplicillium* and *Leptobacillium* was inferred from *rpb1* sequence, based on the Bayesian inference and the maximum likelihood analyses. Each value at a node indicates a bootstrap proportion (the left) and Bayesian posterior probability (the right). The scale bar 0.07 indicates the number of expected mutations per site. The species in bold black font of the *Simplicillium* and *Leptobacillium* were from this study. *B.bassiana* ARSEF 1564 and *B.brongniartii* ARSEF 617 were designated as outgroups.

Using single gene fragments of ITS, nrSSU, nrLSU, *tef-1α* and *rpb1* were used to construct *Simplicillium* and *Leptobacillium* phylogenetic trees, respectively. *Beauveriabassiana* ARSEF 1564 and *B.brongniartii* ARSEF 617 were employed as outgroups (Table 1). Among them, the ITS matrix had 64 sequences, 711 bp of bases, including 783 columns, 381 distinct patterns, 218 parsimony-informative, 64 singleton sites, 500 constant sites. The Best-fit model of the ML tree constructed by the ITS matrix was TIM2+F+I+G4 and the BI tree was GTR+F+I+G4 (Fig. 1). The nrSSU matrix consisted of 21 sequences, 1,122 bp of bases, 2,333 columns, 163 distinct patterns, 30 parsimony-informative, 48 singleton sites and 2,255 constant sites. The Best-fit model for building the nrSSUML tree was TIM3e+I and the BI tree was SYM+I (Fig. 2). The nrLSU had 52 sequences with 1,126 columns, 325 distinct patterns, 91 parsimony-informative, 340 singleton sites, 695 constant sites and 1,019 bp bases. Based on ML and BI, the Best-fit models used to construct the nrLSU phylogenetic framework were K2P+R5, GTR+F+G4, respectively (Fig. 3). The *tef-1α* matrix consists of 52 sequences, 1,090 columns, 431 distinct patterns, 289 parsimony-informative, 82 singleton sites, 719 constant sites and 1,154 bp bases. The Best-fit model of the ML tree constructed by the *tef-1α* matrix was TIM3+F+R8 and the BI tree was GTR+F+I+G4 (Fig. 4). The *rpb1* matrix consisted of 20 sequences, 803 bp bases, 2,971 columns, 390 distinct patterns, 294 parsimony-informative, 113 singleton sites and 2,564 constant sites. Based on ML and BI, the Best-fit models used to construct the *rpb1* phylogenetic framework were TIM2e+I+G4, SYM+I+G4, respectively (Fig. 5). The tree shapes constructed, based on ML and BI, were basically the same and the topological structure adopted in this study was a phylogenetic tree constructed by the maximum likelihood method (Figs 1–5).

**Table 1.** Relevant species information and GeneBank accession numbers for phylogenetic research in this study.

Species	Strain	ITS	nrSSU	nrLSU	*tef-1α*	*rpb1*	*rpb2*	Reference
*Beauveriabassiana*	ARSEF 1564	HQ880761	-	AF373871	HQ880974	HQ880833	HQ880905	Rehner et al. (2011)
*Beauveriabrongniartii*	ARSEF 617	HQ880782	AB027335	-	HQ880991	HQ880854	HQ880926	Rehner et al. (2011)
*Leptobacilliumcavernicola*	LRMH C212	OM622523	OM628842	OM628781	OM654332	OM677781	OM654321	Leplat et al. (2022)
*Leptobacilliumcavernicola*	LRMH C216	OM622524	OM628843	OM628782	OM654333	OM677782	OM654322	Leplat et al. (2022)
*Leptobacilliumchinense*	CGMCC 3.14969	JQ410323	-	JQ410321	-	-	-	Okane et al. (2020)
*Leptobacilliumchinense*	CGMCC 3.14970	JQ410324	-	JQ410322	-	-	-	Okane et al. (2020)
*Leptobacilliumcoffeanum*	COAD 2057	MF066034	-	MF066032	-	-	-	Okane et al. (2020)
*Leptobacilliumcoffeanum*	COAD 2061	MF066035	-	MF066033	-	-	-	Okane et al. (2020)
*Leptobacilliumfiliforme*	URM 7918	-	-	MH979399	-	-	-	Okane et al. (2020)
*Leptobacilliumlatisporum*	TBRC 16288	OP856540	OP850838	OP856529	-	-	-	Preedanon et al. (2023)
*Leptobacilliumleptobactrum*	ZJ14B02	PP385689	-	PP381743	-	-	-	Unpublished
*Leptobacilliumleptobactrum*	AH17C05	PP384754	-	PP380808	-	-	-	Unpublished
Leptobacilliumleptobactrumvar.calidius	CBS 703.86	EF641866	EF641850	KU382226	-	-	-	Zare and Gams (2016)
Leptobacilliumleptobactrumvar.leptobactrum	CBS 771.69	EF641868	EF641852	KU382224	-	-	-	Zare and Gams (2016)
***Leptobacilliumlongiphialidum***	**YFCC 23039272^T^**	**PQ509282**	**PQ508806**	**PQ508808**	**PQ560997**	**PQ567240**	-	**This study**
***Leptobacilliumlongiphialidum***	**YFCC 24079491**	**PQ509281**	**PQ508805**	**PQ508807**	**PQ560996**	**PQ567239**	-	**This study**
*L.marksiae*	BRIP 70307a	PQ061114	-	PQ047739	-	-	-	Tan and Bishop-Hurley (direct submission)
*Leptobacilliummuralicola*	CGMCC 3.19014	MH379983	-	MH379997	-	-	-	Sun et al. (2019)
*Leptobacilliummuralicola*	CGMCC 3.19015	MH379985	-	MH379999	-	-	-	Sun et al. (2019)
*Leptobacilliumsymbioticum*	NBRC 113865	LC485673	-	LC506046	-	-	-	Okane et al. (2020)
*Leptobacilliumsymbioticum*	OPTF00168	LC485675	-	LC506047	-	-	-	Okane et al. (2020)
*Simplicilliumalbum*	LC12442	-	-	-	MK336068	-	-	Zhang et al. (2021)
*Simplicilliumaogashimaense*	JCM 18167	AB604002	-	LC496874	LC496904	-	-	Nonaka et al. (2013)
*Simplicilliumaogashimaense*	JCM 18168	AB604004	-	LC496875	-	-	-	Nonaka et al. (2013)
*Simplicilliumaraneae*	DY101811	OM743774	-	OM743792	OM818465	-	-	Chen et al. (2022)
*Simplicilliumaraneae*	DY101812	OM743840	-	OM743846	OM818466	-	-	Chen et al. (2022)
*Simplicilliumcalcicola*	LC5586	KU746706	-	KU746752	KX855252	-	-	Zhang et al. (2017)
*Simplicilliumcalcicola*	LC5371	KU746705	-	KU746751	KX855251	-	-	Zhang et al. (2017)
*Simplicilliumcicadellidae*	GY11012	MN006244	-	-	MN022264	MN022272	-	Chen et al. (2019)
*Simplicilliumcicadellidae*	GY11011	MN006243	-	-	MN022263	MN022271	-	Chen et al. (2019)
*Simplicilliumcoccinellidae*	DY101791	MT453861	MT453863	-	MT471341	-	-	Chen et al. (2021)
*Simplicilliumcoleopterorum*	SD05381	OM743920	-	OM743925	OM818467	-	-	Chen et al. (2022)
*Simplicilliumcoleopterorum*	SD05382	OM744109	-	OM744170	OM818468	-	-	Chen et al. (2022)
*Simplicilliumcylindrosporum*	JCM 18169	AB603989	-	LC496876	LC496906	-	-	Nonaka et al. (2013)
*Simplicilliumcylindrosporum*	JCM 18170	AB603994	-	LC496877	LC496907	-	-	Nonaka et al. (2013)
*Simplicilliumformicae*	DY09641	OR121054	-	OR121057	OR126571	-	-	Unpublished
*Simplicilliumformicae*	DY09642	OR121055	-	OR121056	OR126572	-	-	Unpublished
*Simplicilliumguizhouense*	DY10051	OM743225	-	OM743226	OM818453	-	-	Chen et al. (2022)
*Simplicilliumguizhouense*	DY10052	OM743241	-	OM743252	OM818454	-	-	Chen et al. (2022)
*Simplicilliumhumicola*	LC 12494	-	-	-	MK336072	-	-	Zhang et al. (2021)
*Simplicilliumhumicola*	CGMCC 3.19573	NR_172845	-	MK329041	MK336071	-	-	Unpublished
*Simplicilliumhymenopterorum*	DY101692	MT453851	-	-	MT471338	-	-	Unpublished
*Simplicilliumhymenopterorum*	DY101691	MT453848	MT453849	-	MT471337	MT471344	-	Unpublished
*Simplicilliumlamellicola*	JC-1	MT807906	MT807908	MT807907	-	-	-	Unpublished
*Simplicilliumlamellicola*	CBS 116.25	AJ292393	-	-	DQ522356	DQ522404	DQ522462	Nonaka et al. (2013)
*Simplicilliumlanosoniveum*	CBS 704.86	AJ292396	-	-	DQ522358	DQ522406	DQ522464	Nonaka et al. (2013)
*Simplicilliumlarvatum*	DY101731	OM743438	-	OM743441	OM818462	OM818460	-	Chen et al. (2022)
*Simplicilliumlepidopterorum*	GY29132	MN006245	-	-	MN022266	MN022274	-	Chen et al. (2019)
*Simplicilliumlepidopterorum*	GY29131	MN006246	-	-	MN022265	MN022273	-	Chen et al. (2019)
*Simplicilliumminatense*	JCM 18176	AB603992	LC496893	LC496878	LC496908	-	-	Nonaka et al. (2013)
*Simplicilliumminatense*	JCM 18178	AB603993	LC496894	LC496879	LC496909	-	-	Nonaka et al. (2013)
*Simplicilliumneolepidopterorum*	DY101752	MT453857	-	-	MT471340	-	-	Chen et al. (2021)
*Simplicilliumneolepidopterorum*	DY101751	MT453854	MT453856	-	MT471339	-	-	Chen et al. (2021)
*Simplicilliumobclavatum*	CBS 311.74	AJ292394	-	AF339517	EF468798	-	-	Nonaka et al. (2013)
*Simplicilliumobclavatum*	SUF81	-	-	MK788174	-	-	-	Unpublished
*Simplicilliumpechmerlense*	CBS 147188	MW031272	-	MW031268	MW033224	MW033222	-	Leplat et al. (2021)
***Simplicilliumpuwenense***	**YFCC 23129490^T^**	**PQ508796**	**PQ508799**	**PQ508802**	**PQ537122**	**PQ560994**	-	**This study**
***Simplicilliumpuwenense***	**YFCC 23089322**	**PQ508797**	**PQ508800**	**PQ508803**	**PQ537123**	-	-	**This study**
***Simplicilliumpuwenense***	**YFCC 23069492**	**PQ508798**	**PQ508801**	**PQ508804**	**PQ537124**	**PQ560995**	-	**This study**
*Simplicilliumscarabaeoidea*	DY101392	MT453845	-	-	MT471336	-	-	Chen et al. (2021)
*Simplicilliumscarabaeoidea*	DY101391	MT453842	MT453843	-	MT471335	MT471343	-	Chen et al. (2021)
*Simplicilliumsinense*	AFMCCC 16a	OQ332403	-	-	OQ352167	-	-	Yan et al. (2023)
*Simplicilliumsinense*	AFMCCC 16b	OQ332404	-	-	OQ352168	-	-	Yan et al. (2023)
*Simplicilliumspumae*	JCM 39054	LC496871	-	LC496887	LC496917	-	-	Kondo et al. (2020)
*Simplicilliumspumae*	JCM 39050	LC496869	LC496898	LC496883	LC496913	-	-	Kondo et al. (2020)
*Simplicilliumsubtropicum*	JCM 18180	AB603990	-	LC496880	LC496910	-	-	Nonaka et al. (2013)
*Simplicilliumsubtropicum*	JCM 18181	AB603995	-	LC496881	LC496911	-	-	Nonaka et al. (2013)
*Simplicilliumsympodiophorum*	JCM 18184	AB604003	-	LC496882	LC496912	-	-	Nonaka et al. (2013)
*Simplicilliumyunnanense*	YFCC 7134	-	MN576729	MN576785	MN576955	MN576845	-	Wang et al. (2020)
*Simplicilliumyunnanense*	YFCC 7133	-	MN576728	MN576784	MN576954	MN576844	-	Wang et al. (2020)

Based on the phylogenetic framework constructed by single gene fragments, it was found that the resulting topologies were roughly similar and there was no obvious conflict between different gene fragments. The species *S.puwenense* and *L.longiphialidum* collected and described in this study were located in roughly the same position in each phylogenetic tree, forming monophyletic, with high support rate and stable topological structure. In the topology constructed, based on ITS, nrLSU and *tef-1α* matrices, *S.puwenense* and *S.formicae* D.P. Wei & K.D. Hyde were closely related. In phylogenetic trees constructed by ITS and nrLSU matrices, *L.longiphialidum* and *L.marksiae* Tan, Bishop-Hurley & Marney came together.

#### ﻿Phylogenetic tree reconstructed from multi-gene combined dataset

**Figure 6.**
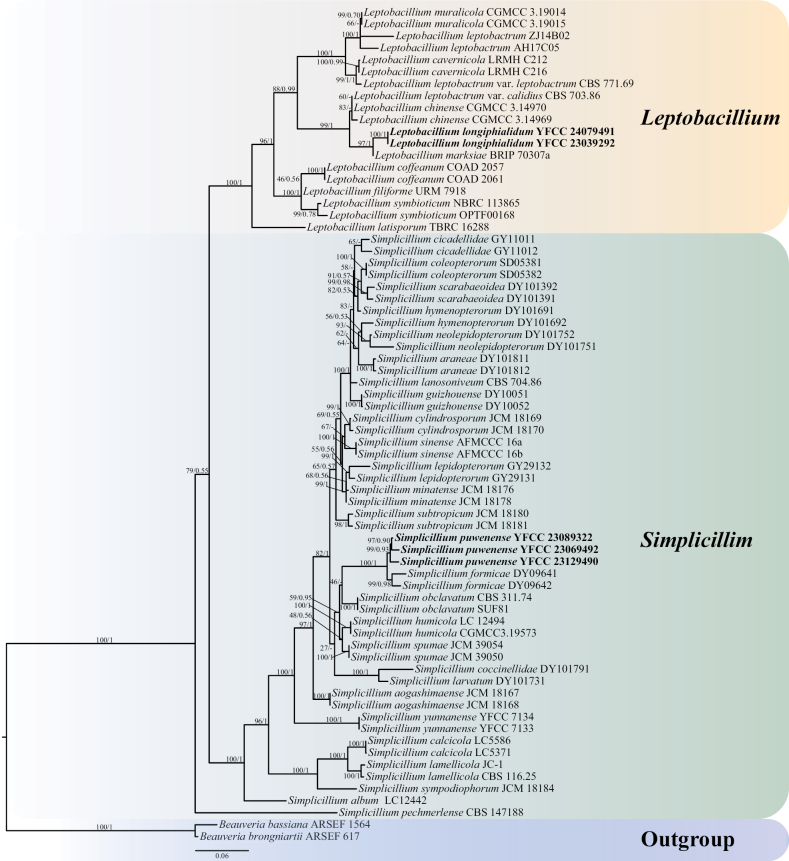
The phylogenetic tree of *Simplicillium* and *Leptobacillium* was inferred from six-gene dataset (ITS, nrSSU, nrLSU, *tef-1α*, *rpb1*, *rpb2*), based on the Bayesian inference and the maximum likelihood analyses. Each value at a node indicates a bootstrap proportion (the left) and Bayesian posterior probability (the right). The scale bar 0.06 indicates the number of expected mutations per site. The species in bold black font of the *Simplicillium* and *Leptobacillium* were from this study. *B.bassiana* ARSEF 1564 and *B.brongniartii* ARSEF 617 were designated as outgroups.

The phylogenetic framework for the genera *Simplicillium* and *Leptobacillium*, comprising 70 taxonomic units, was constructed, based on a six-gene dataset utilising the maximum likelihood method and Bayesian inference. *B.bassiana* ARSEF 1564 and *B.brongniartii* ARSEF 617 were employed as outgroups (Table 1). The joint matrix comprised 14,494 columns, 1,873 distinct patterns, 1,190 parsimony-informative, 770 singleton sites and 12,534 constant sites. The most appropriate model for the ML analysis amongst the 286 models simulated by ModelFinder was TIM2+F+R10, which achieved an IQ-TREE best score of -32893.153 and a Total tree length of 2.122. The parameters of the TIM2+F+R10 model used to analyse the dataset were estimated, based on the following nucleotide frequencies: A = 0.243, C = 0.262, G = 0.261, T = 0.233, A–C = 1.15866, A–G = 2.32357, A–T = 1.15866, C–G = 1.00000, C–T = 5.27826 and G–T = 1.00000. The GTR+F+I+G4 model was determined as the most suitable model for the BI analysis using ModelFinder amongst the 24 simulated models. It achieved an IQ-TREE best score of -33090.992 and a total tree length of 1.634. The phylogenetic trees constructed using the maximum likelihood (ML) and the Bayesian inference (BI) methods exhibited a high degree of similarity, as depicted in Fig. 6.

The phylogenetic tree of the six-gene joint dataset revealed that the majority of species were grouped in distinct branches with robust support, indicating a stable topology (Fig. 6). The strains YFCC 23129490, YFCC 23069492 and YFCC 23089322, collected and described in this study, formed a well-supported single branch. *S.puwenense* and *S.formicae* were identified as sister species, constituting an independent clade with BP and BPP values of 100% and 1, respectively, while maintaining topological stability. YFCC 24079491 and YFCC 23039272 clustered together (BP = 100%, BPP = 1). *L.longiphialidum* and *L.marksiae* clustered into a clade, with BP and BPP of 97% and 1, respectively, forming sister species and receiving high support.

### ﻿Taxonomy

#### ﻿ Simplicillium puwenense

Taxon classificationFungiHypocrealesCordycipitaceae

Hong Yu bis, Y.L. Lu & Jing Zhao sp. nov.

211C5CBC-66DC-5890-85C0-4CD96E631094

856314

Fig. 7

##### Etymology.

Named after the location Puwen Town where the pattern material was collected.

##### Holotype.

China • Yunnan Province, Xishuangbanna Dai autonomous prefecture, Jinghong City, Puwen Town. Specimens were collected from an evergreen broad-leaved forest, alt. 1,062 m, 100°58'60"E, 22°31'20"N, 13 December 2023, Hong Yu (***holotype***: YHH SP2312001, ***ex-type living culture***: YFCC 23129490).

##### Description.

***Sexual morph*.** Not found.

***Asexual morph*.** Colonies on PDA medium moderate growth,diameter of 32–35 mm at 25 °C for 14 days, convex in middle surface, white fluffy to cotton like, dense, octahedral crystals absent, reverse brown to light brown with radial emission grooves. Hyphae septate, branched, transparent, with a diameter of 0.67–1.76 µm and smooth-walled. Cultures readily produced phialides and conidia after 14 days on PDA medium at room temperature. Phialides arising were slender, solitary, rod-shaped or columnar, measuring 3.37–52.57 µm in length and 0.5–1.6 µm in width. Conidia, transparent, single celled, smooth-walled, elliptical or oval or cylindrical, 1.19–2.41 × 0.88–1.6 µm. The conidia aggregated into a spherical shape at the top of the phialides, with a size of approximately 3.59–6.59 × 2.6–6.7 µm.

**Figure 7.**
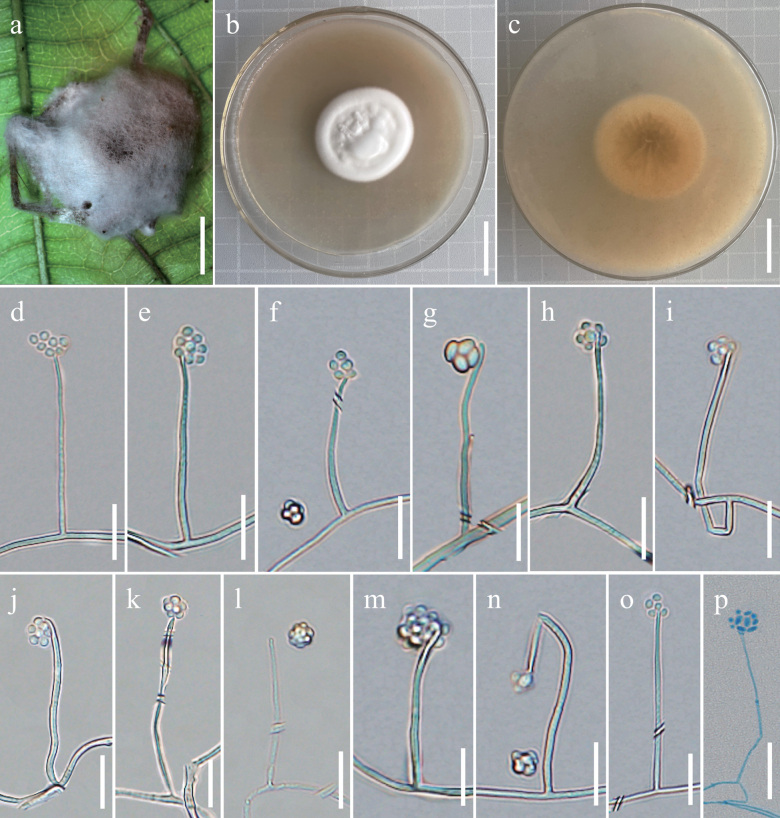
Morphology of *Simplicilliumpuwenense***a** wild material **b** colonies obverse in PDA at 25 °C **c** colonies reverse on PDA at 25 °C **d–p** phialides bearing conidia. Scale bars: 3 mm (**a**); 3 cm (**b, c**); 10 µm (**d–f**); 8 µm (**g**); 10 µm (**h, i**); 12 µm (**j**); 10 µm (**k, l**); 8 µm (**m**); 10 µm (**n, o**); 15 µm (**p**).

##### Host.

Spider.

##### Distribution.

China, Yunnan Province.

##### Additional material examined.

China • Yunnan Province, Honghe Hani and Yi autonomous prefecture, the Xilong Mountains. Specimens were collected from an evergreen broad-leaved forest, alt. 1,715 m, 102°32'48"E, 22°45'20"N, 1 June 2023, Jing Zhao (paratype: YHH SP2306001, ex-paratype living culture: YFCC 23069492); • Puwen Town,collected from an evergreen broad-leaved forest, alt. 1,019 m, 100°58'42"E, 22°31'10"N, 4 August 2023, Hong Yu (Specimen number: YHH SP2308001, Strain number: YFCC 23089322).

##### Remarks.

Phylogenetically, three samples of *S.puwenense* were grouped together on a single branch, forming a monophyletic clade. It was identified as the sister species to *S.formicae*, supported by robust statistical evidence from both the Bayesian inference (BPP = 1) and the maximum likelihood analysis (BP = 100%). Both *S.puwenense* and *S.formicae* exhibited a stable topological structure with BP and BPP values of 100%. Morphologically, the surface of *S.puwenense* appeared centrally convex and exhibited a white, fluffy or cotton-like texture with densely arranged radial emission grooves ranging from reverse brown to light brown. Additionally, the conidia were observed to aggregate into spherical clusters at the apex of phialides (Table 2).

**Table 2.** Morphological comparisons of asexual morphs in the genus *Simplicillium*.

Species	Colony on PDA	Phialides (µm)	Conidia (µm)	Octahedral crystals	References
*S.album*	White, with a yellowish discharge, reverse beige to thick yellow, fluted	2–3 whorls or Solitary, 13.0–40.0 × 1.5–3.0 µm	Two conidia: macroconidia sickle-shaped or fusiform, 8.0–11.0 (–13.0) × 2.0–3.5 µm; Microconidia oval or oblong, 3.0–4.0 × 1.5–2.0 µm	Present	Zhang et al. (2021)
*S.aogashimaense*	White, reverse yellow white	Solitary, a few 2–3 whorls, slender and long (19–) 23–53 × 1.2–2.0 µm	Cylindrical, 4.2–6.5 × 1.2–2.0 (–2.3), conidia aggregate into spherical small heads at the top of bottle stem	Present	Nonaka et al. (2013)
*S.araneae*	White fluff, reverse yellow to brown	Solitary, slender, tapering from base to top, 32.9–47.1 × 1.2–2.4 µm	Subspherical, spherical, or elliptical, 1.8–2.9 × 1.2–1.8 µm	Absent	Chen et al. (2022)
*S.calcicola*	White or yellow, reverse light yellow to yellow	2–3 whorled or solitary, 14.0–38.0 × 1.0–2.0 µm	Two conidia: macroconidial fusiform, 4.5–8.0 × 1.0–2.0 µm; microconidia oval or globose or spherical, 2.0–3.5 × 1–1.5 µm	Absent	Zhang et al. (2017)
*S.cicadellidae*	White, reverse yellow	Solitary, 12.9–18.3 × 0.8–1.1 µm	Ellipsoid, 1.8–2.8 × 1.4–1.8 µm	Absent	Chen et al. (2019)
*S.coccinellidae*	White fluff, reverse yellow to light brown	Solitary, 4.9–62.1 × 1.0–1.5 µm	Subspherical or cylindrical or elliptical, 2.0–3.4 × 1.6–2.0 µm	Absent	Chen et al. (2021)
*S.coleopterorum*	White fluff, reverse light brown to brown	Solitary, 34.5–64.1 × 0.7–1.2 µm	Spherical or subspherical or elliptical, 2.1–3.3 × 1.5–1.9 µm	Absent	Chen et al. (2022)
*S.cylindrosporum*	White, reverse blond	2–3 whorled or solitary, 17–32 × 1.2–2.0 (–2.5) µm	Spherical or cylindrical, 3.0–4.5(–5.0) × 1.0–2.0 µm	Present	Nonaka et al. (2013)
*S.formicidae*	White, reverse light brown to brown, brown secretions	Solitary, 51–70.1 × 0.7–0.9 µm	Conidia aggregate into spherical slimy heads, mostly filamentous or fusiform, 3.9–7.9 × 0.8–1.3 µm	Absent	Chen et al. (2019)
*S.guizhouense*	White, reverse yellow to light yellow	Solitary, 1.1–52.2 × 1.0–1.8 µm	Oval or spherical, 2.4–2.9 × 1.6–1.8 µm	Absent	Chen et al. (2022)
*S.humicola*	White, light-yellow secretions, reverse light yellow to brown	2–3 whorled or solitary, 20.0–35.0 (–47.0) × 1.5–3.0 µm	Oblong or oval, 3.0–5.0 × 1.5–3.0 µm	Present	Zhang et al. (2021)
*S.hymenopterorum*	White, reserve light yellow	Mainly solitary, rarely whorls, 19.3–46.2 × 1.1–2.3 µm	Cylindrical to subellipsoidal, 2.1–2.8 × 1.3–1.9 µm, forming a subspherical small head at the top of the stem	Absent	Chen et al. (2021)
*S.lamellicola*	White, reserve light yellow	15–50 × 0.7–1.0 µm	Two conidia: macroconidia fusiform, 4.5–9.0 × 0.8–1.0 µm; microconidia ovoid to ellipsoid, 2.0–3.0 × 0.7–1.2 µm	Present	Zare and Gams (2001)
*S.lanosoniveum*	White or cream, reverse brownish cream to light yellow	Solitary, 20.0–40.0 × 1.1–2.0 µm	Spherical or ellipsoidal, 2.0–4.5 × 1.0–3.0 µm, forming a spherical or ellipsoidal tip at the top of the phialides,		Wei et al. (2019)
*S.lepidopterorum*	White, reserve light yellow	Solitary, 15.3–26.2 × 0.7–1.4 µm	Spindle-shaped or oval, 1.6– 2.4 × 1.4–1.7 µm, forming a slimy spherical head at the top of the phialides	Absent	Chen et al. (2019)
*S.minatense*	White, no secretion, reverse brown	Mainly solitary, rarely in whorls of 2–3, 11.0–31.0 (–47.0) × 1.0–1.7 µm	Spherical, 2.0–3.5 × 1.8–2.5 (–2.8) µm, forming a subglobose or ellipsoidal tip at the top of the phialides	Present	Nonaka et al. (2013)
*S.neolepidopterorum*	White, reverse yellow to light yellow	Solitary, 34.1–44.3 × 1.0–1.7 µm Solitary, 34.1–44.3 × 1.0–1.7 µm	Solitary, ellipsoidal to cylindrical, occasionally in short imbricate chains, 2.5–3.8 × 1.5–2.1 µm	Absent	Chen et al. (2021)
*S.niveum*	White	2–5 whorled, 10–20.5 (25.0) × 1–2 µm	Top growth, elongated or elliptical in shape, 3.0–4.5 (–6) × 1–2 µm		Crous et al. (2021)
*S.pechmerlense*	White, reverse light yellow to orange	Solitary, 16.0–31.0 × 0.9–1.2 µm	Two conidia: macroconidia fusiform, 5.0–8.0 × 1–1.6 µm; microconidia subglobular or elliptic, 1.8–3.0 × 0.9–1.5 µm, forming a slimy spherical head at the top of the phialides,	Absent	Leplat et al. (2021)
***S.puwenense***	**White fluffy to cotton like, convex in middle surface, reverse brown to light brown with radial emission grooves**	**Slender, solitary, rod-shaped or columnar, measuring 3.37–52.57 µm in length and 0.5–1.6 µm in width**	**Elliptical or oval or cylindrical, 1.19–2.41 × 0.88–1.6 µm. forming a spherical shape at the top of the phialides, 3.59–6.59 × 2.6–6.7 µm in size**	**Absent**	**This study**
*S.scarabaeoidea*	White, reverse light yellow	Solitary, 18.5–63.4 × 1.1–1.4 µm	Ellipsoidal, 1.9–2.9 × 1.4–2.0 µm	Absent	Chen et al. (2021)
*S.subtropicum*	White, reverse brownish orange to brown	(15.0–) 20–42 (–50.0) × 1.0–2.3 µm; Solitary, rarely in whorls of 2–3, (15.0–) 20.0–42.0 (–50.0) × 1.0–2.3 µm	Subglobose or ellipsoid, 2.3–4.0 (–4.5) × 1.5–3.3 µm, forming a spherical tip at the top of the phialides, 2.3–4.0 (–4.5) × 1.5–3.3 µm in size	Present	Nonaka et al. (2013)
*S.sympodiophorum*	White, reverse yellow white	2–4 whorled or solitary, 20.0–34 (–47.0) × 0.5–1.3 µm	Oval to ellipsoidal, 2.2–3.5 × 1.0–2.0 µm	Present	Nonaka et al. (2013)
*S.yunnanense*	White to light yellow, grayish orange to brown on back	Solitary, 5.8–16.9 × 1.1–1.5 µm	Cylindrical, 2.5–3.4 × 0.7–1.1 µm, conidia usually form chains at the top of the phialides	-	Wang et al. (2020)

#### ﻿ Leptobacillium longiphialidum

Taxon classificationFungiHypocrealesCordycipitaceae

Hong Yu bis, Y.L. Lu & Jing Zhao sp. nov.

26F92A1F-34E3-57FB-A8CD-FD93B81DA713

856313

Fig. 8

##### Etymology.

Referring to its longer phialides than those of the close relationship species in this genus.

##### Holotype.

China • Hainan Province, Qiongzhong City, Limushan Town, Limushan National Forest Park. Specimens were collected from an evergreen broad-leaved forest, alt. 589.9 m, 109°44'28"E, 19°10'41"N, 8 March 2023, Jing Zhao (***holotype***: YHH LL2303001, ***ex-type living culture***: YFCC 23039272).

##### Description.

***Sexual morph*.** Not found.

***Asexual morph*.** The colony was incubated at 25 °C on PDA medium for 14 days, the growth rate was slow, the diameter was 25–27 mm, the middle was fluffy to cotton, dense, convex and radial wrinkles, white and reverse brown to light yellow on the back. Mycelium branches, smooth walls, septate, transparent, with a diameter of approximately 0.97 × 1.72 µm. Cultures readily produced phialides and conidia after 10 days on PDA medium at room temperature. Phialides solitary, columnar, tapering from base to apex, 24.01–205.77 µm long, 1.00–2.24 µm wide. Conidia 2.88–4.54 × 1.18–1.95 µm, transparent, single celled in chains, smooth walls, narrow columnar or spindle-shaped, with apical conidia elliptical or nearly spherical in shape.

**Figure 8.**
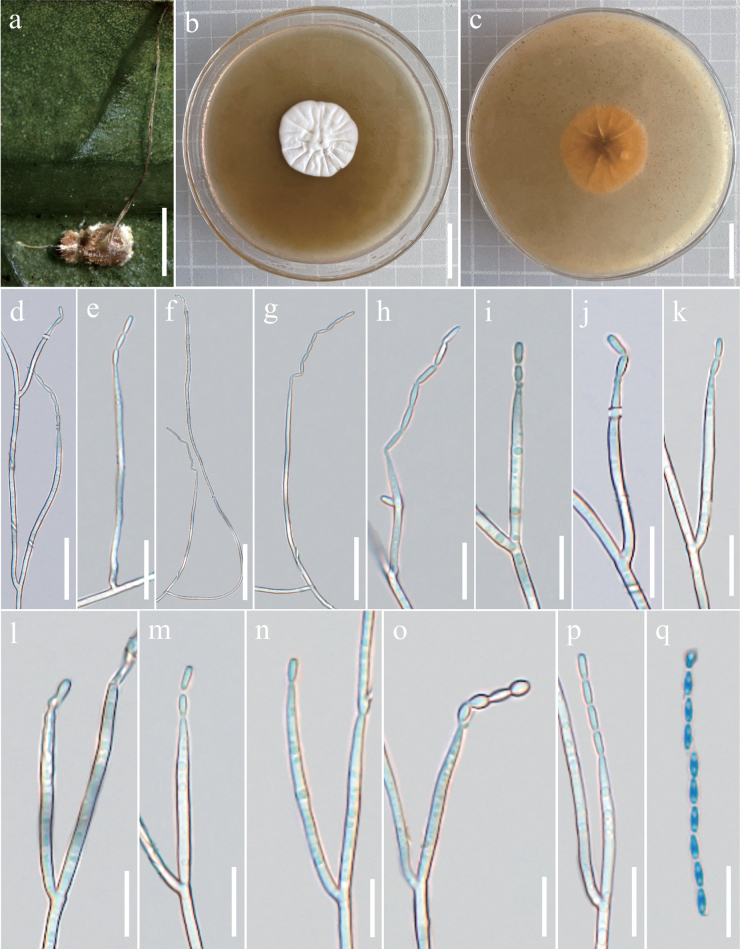
Morphology of *Leptobacilliumlongiphialidum***a** wild material **b** colonies obverse in PDA at 25 °C **c** colonies reverse on PDA at 25 °C **d–p** phialides bearing conidia **q** conida. Scale bars: 2 mm (**a**); 2 cm (**b, c**); 20 µm (**d**); 12 µm (**e**); 30 µm (**f**); 20 µm (**g**); 10 µm (**h–k**); 9 µm (**l**); 10 µm (**m**); 7 µm (**n**); 8 µm (**o**); 10 µm (**p, q**).

##### Host.

Spider.

##### Distribution.

China, Hainan Province, Guangdong Province.

##### Additional material examined.

China • Guangdong Province, Huizhou City, Boluo County, 511 Township Road. Specimens were collected from an evergreen broad-leaved forest, alt. 29.4 m, 114°24'5"E, 23°14'32"N, 23 July 2024, Hong Yu and Y.L. Lu (paratype: YHH LL2407001, ex-paratype living culture: YFCC 24079491.

##### Remarks.

The key characteristic of *L.longiphialidum* was its independent, columnar shape and the presence of narrow or fusiform spores. Phylogenetic analyses showed that *L.longiphialidum* belonged to the *Leptobacillium* clade and was closest to *L.marksiae*. However, the host and collection sites of *L.longiphialidum* were spiders and China, respectively and the host and collection sites of *L.marksiae* were an unidentified dead insect and Queensland, Australia, respectively. *L.longiphialidum* and *L.marksiae* were distinguished by genetic distance. (Table 3).

**Table 3.** Morphological comparisons of asexual morphs in the genus *Leptobacillium*.

Species	Colony on PDA	Phialides (µm)	Conidia (µm)	References
*L.cavernicola*	White, reverse usually dark brown	Mainly solitary, slender, tapering toward tip, 5.1–27.2 × 1.2–1.7 µm	Forming long, slender chains, narrowly cylindrical to slightly fusiform, some were slightly lemon-shaped, first-formed conidium were usually shorter, obovoid to pyriform with a rounded distal end, 3.1–6.9 × 0.9–1.5 µm	Leplat et al. (2022)
*L.chinense*	White, reverse cream to light yellow	Solitary, (6.0–) 15–30 (–68.0) × 1.5 µm	Ellipsoidal or oval or cylindrical, 3.5–5.0 × 1.0–1.5 µm, the conidia aggregate into chains, with the apex conidia subspherical or obovoid, 1.5–2.5 × 1.5–2.0 µm	Liu and Cai (2012)
*L.coffeanum*	White, reverse cream	Solitary, few 2–3 whorls, 11.0–44.0 (–70.0) × 1.0–2.4 µm	Two conidia, macroconidia spindle-shaped, 5.3–8.8 × 1.0–1.6 µm; microconidia oval to fusiform, 2.2–3.8 × 0.8–1.5 µm	Gomes et al. (2018)
*L.filiforme*	White, reverse light yellow	Solitary, 9.0–18.0 × 1.0 µm	Fusiform to filamentous, chained, sometimes forming zigzag chains, 7.2–12.5 × 1.0 µm	Crous et al. (2018)
*L.latisporum*	White, reverse greyish orange to orange white	13.2–40.8 × 2.9–4.8 μm	Shuttle shaped to narrow cylindrical, with single cells forming long chains, 3.9–6.3 × 1.9–3.9 μm	Preedanon et al. (2023)
***L.longiphialidum***	**White, reverse brown to light yellow**	**Solitary, 24.01–205.77 × 1.00–2.24 µm**	**Narrow columnar or spindle shaped, 2.88–4.54 × 1.18–1.95 µm, single celled in chains, with apical conidia elliptical or nearly spherical in shape**	**This study**
L.leptobactrumvar.calidius	White to cream, reverse Light yellow to brown	Solitary, few 1–2 whorls, 18.4–60.0 × 0.7–2.0 µm	Narrow cylindrical (rod-shaped) to slightly fusiform, 3.0–5.7 × 0.7–1.7 µm	Zare and Gams (2016)
L.leptobactrumvar.leptobactrum	White to cream, reverse Light yellow to yellowish brown	15.8–31.7 × 0.7–1.5 µm Solitary, few 2–3 whorls, 15.8–31.7 × 0.7–1.5 µm	Narrow rod-shaped or narrow cylindrical (rod-shaped), 3.0–6.1 × 0.8–2.1 µm	Zare and Gams (2016)
*L.leptobactrum*	White, gray white to pinkish white, reverse orange to orange brown, gray white, light yellow, milky white to dark yellow	Solitary, few 1–2 branches, 20.0–45.0 µm long, Base width 1–2 µm, top width 0.5–0.7 µm	Narrow cylindrical (rod-shaped) to slightly fusiform, 4.5–8.0 × 0.8–1.5 (–2.0) µm	Zare and Gams (2016)
*L.muralicola*	White, gray white to green white, reverse light yellow, milky white to dark yellow, orange to orange brown, ochraceous	Solitary, few 1–2 branches, 20.0–45.0 µm long, Base width 1.0–2.0 µm, top width 0.5–0.7 µm	Narrow cylindrical (rod-shaped) to slightly fusiform, 4.5–6.0 × 1.0–2.0 µm	Sun et al. (2019)
*L.symbioticum*	White, reverse orange yellow to orange-brown	Solitary, few 2–3 whorls, 7.1–30.6 × 1.6–3.5 µm	Slightly fusiform to narrowly cylindrical, 4.0–6.9 × 0.7–1.6 µm	Okane et al. (2020)

## ﻿Discussion

The genera of *Simplicillium* and *Leptobacillium* were found to be the most closely related within the family of Cordycipitaceae. They exhibited a wide distribution and were commonly observed on various substrates or hosts, including air, seawater, rocks, leaves, soil, insects, fungi, freshwater environments, murals, rocks and caves (Liu and Cai 2012; Zare and Gams 2016; Crous et al. 2018; Gomes et al. 2018; Sun et al. 2019; Wei et al. 2019; Kondo et al. 2020; Okane et al. 2020; Wang et al. 2020; Leplat et al. 2021). Chen et al. (2019) first reported insect-associated species of *Simplicillium* while later reporting an additional eight arthropod-related species of the genus *Simplicillium* (Chen et al. 2021, 2022). Furthermore, Leplat et. al (2022) isolated *L.cavernicola* Leplat from caves as a representative species of the genus *Leptobacillium* whereas *L.muralicola* Z. Sun, Qin Y. Ge, Zhi B. Zhu & Xing Z. Liu was isolated from mural paintings in a Koguryo tomb in China (Sun et al. 2019).

The macroscopic and microscopic morphology of most species in the genera of *Simplicillium* and *Leptobacillium* are quite similar and it is difficult to distinguish specific species, based on only morphological features. Thus, it is often necessary to combine morphological and molecular data for species identification. The utilisation of ITS and nrLSU by Liu and Cai (2012) yielded more accurate outcomes in the identification of *Simplicillium* species. To date, multi-site phylogenies incorporating the combined analysis of ribosomal DNA and functional protein-coding genes have been extensively employed in fungal phylogeny research, yielding numerous significant findings (Sung et al. 2007; Luangsa-ard et al. 2017; Mongkolsamrit et al. 2020; Wang et al. 2020). The results showed that the molecular phylogenies of *Simplicillium* and *Lecanicillium*, based on ITS fragment, nrLSU fragment and six-gene combined dataset, were more stable in topology. This was consistent with the results of previous studies. In this study, two novel species, *S.puwenense* and *L.longiphialidum*, were identified and characterised through meticulous morphological examination and rigorous phylogenetic analysis.

Through morphological observation, it was found that phialides of species in the genus of *Simplicillium* were solitary and could be distinguished from those of the genus of *Lecanicillium* (Chen et al. 2021). It was observed that a prominent characteristic of species within the *Simplicillium* genus was the solitary nature of phialides, wherein conidia typically adhered to the apex of phialides in chains exhibiting spherical, sticky, or tile-like properties, ultimately resulting in the formation of octahedral crystals (Zare and Gams 2001). The primary distinguishing feature of *Leptobacillium* species lay in the presence of two conidia; single cells arranged in clusters with near-spherical or elliptical conidia at the apex and other narrow columnar (rod) to fusiform-shaped conidia (Zare and Gams 2016; Leplat et al. 2022). The phialides of *S.puwenense* collected in this study were slender, solitary, rod-shaped or columnar; the conidia were transparent, single-celled with smooth walls and had an oval or cylindrical shape. They formed aggregates into a spherical structure at the apex of the phialides. These characteristics aligned closely with the primary identification features described for *Simplicillium* species by Zare and Gams (2001). The phialides of *L.longiphialidum* appeared as solitary and columnar structures. Two types of conidia were observed, i.e. one type consisted of single cells clustered together in chains, while the other type was oval or nearly spherical and located at the apex. Additionally, there was another type of narrow columnar or spindle-shaped conidium present, which was consistent with previous studies on *Leptobacillium* species (Zare and Gams 2016; Leplat et al. 2022).

In phylogenetic trees, most species of the genera *Simplicillium* and *Leptobacillium* were clustered in their separate clades and were well supported and topologically stable. However, the phylogenetic framework showed that two samples of *L.leptobactrum*, ZJ14B02 and AH17C05, did not form a monophyletic clade. The ITS sequence and nrLSU sequence of strain ZJ14B02 contained 547 bp and 909 bp, respectively. The ITS and nrLSU sequences of strain AH17C05 contained 557 bp and 929 bp, respectively. It was found that the head and tail bases of ITS sequence of samples ZJ14B02 and AH17C05 were different from those of nrLSU sequences. It was speculated that the two samples of *L.leptobactrum* did not form a monophyletic clade, which might be caused by the poor processing of the fore-tail primer sequence. Zare and Gams (2016) initially described *L.leptobactrum*, composed of L.leptobactrumvar.leptobactrum and L.leptobactrumvar.calidius, which were distinguished by their optimal growth temperature. The optimum temperature for growth of L.leptobactrumvar.leptobactrum was 18–21 °C, no growth at 30 °C (Zare and Gams 2016). The optimum temperature for growth of L.leptobactrumvar.calidius was 24–27 °C, reduced growth at 30 °C, no growth at 33 °C (Zare and Gams 2016). Phylogenetic studies had placed two strains in unexpected clades, namely L.leptobactrumvar.leptobactrum and L.leptobactrumvar.calidius. In the phylogenetic framework constructed by nrSSU, L.leptobactrumvar.leptobactrum and L.leptobactrumvar.calidius clustered into a clade. The findings of phylogenetic frameworks, based on ITS, nrLSU and six-gene datasets, revealed that L.leptobactrumvar.calidius and *L.chinense* formed a cluster, while L.leptobactrumvar.leptobactrum and *L.cavernicola* also clustered together. This was consistent with the findings of Leplat et al. (2022).

*S.pechmerlense* J. Leplat constituted an independent clade that exhibited slight differences compared to the previously studied phylogenetic framework (Leplat et al. 2021). However, it was the same as the phylogenetic framework reconstructed by Chen et al. (2022). Additionally, Leplat et al. (2021) found that the underside of the colony of *S.pechmerlense* was light yellow to orange, the phialides was solitary and there were two kinds of conidium, the macroconidia was spindle, 5.0–8.0 × 1.0–1.6 µm. The microconidia were subspherical or elliptic, 1.8–3.0 × 0.9–1.5 µm, forming slimy globular heads at the top of the phialides. *S.pechmerlense* phialides solitary and conidia attached to the top of the phialides with slimy heads fit the main identification characteristics of *Simplicillium* (Zare and Gams 2001). *S.pechmerlense* was morphologically similar to *S.calcicola* Z.F. Zhang, F. Liu & L. Cai and *S.album* Z.F. Zhang & L. Cai (Leplat et al. 2021). The phialides of *S.calcicola* and *S.album* were 2–3-whorled or solitary (Zhang et al. 2017, 2021), while *S.pechmerlense* were solitary. The solitary phialides could distinguish *S.pechmerlense* from *S.calcicola* and *S.album*. Species of *Simplicillium* have frequently been identified using ITS and nrLSU sequences (Liu and Cai 2012). Phylogenetic analyses, based on single gene fragments revealed an unstable systematic position for *S.pechmerlense*. However, the morphological characteristics of *S.pechmerlense* align with the primary identification features of *Simplicillium*. Consequently, it was determined that *S.pechmerlense* should be retained within the genus *Simplicillium*. The inclusion of supplementary materials, such as morphological data, would be essential for further verification since only one strain of polygenic sequence data was available for L.leptobactrumvar.leptobactrum and L.leptobactrumvar.calidius.

## Supplementary Material

XML Treatment for Simplicillium puwenense

XML Treatment for Leptobacillium longiphialidum

## ﻿Additional information

### Author ORCIDs

Yingling Lu https://orcid.org/0009-0008-8119-1975

Songyu Li https://orcid.org/0009-0007-9589-0892

Zuoheng Liu https://orcid.org/0000-0003-4118-3694

Jing Zhao https://orcid.org/0000-0001-7871-2209

Zhiyong Yu https://orcid.org/0000-0001-8276-5901

Zongli Liang https://orcid.org/0009-0006-5553-8811

Hailong He https://orcid.org/0009-0000-7862-0865

Jianhong Li https://orcid.org/0009-0001-1234-7816

Yun Huang https://orcid.org/0009-0007-3429-1490

Xinming Li https://orcid.org/0009-0001-7198-281X

Hong Yu https://orcid.org/0000-0002-2149-5714

### Data availability

All of the data that support the findings of this study are available in the main text.
